# Domestic Pig Unlikely Reservoir for MERS-CoV

**DOI:** 10.3201/eid2306.170096

**Published:** 2017-06

**Authors:** Emmie de Wit, Friederike Feldmann, Eva Horne, Cynthia Martellaro, Elaine Haddock, Trenton Bushmaker, Kyle Rosenke, Atsushi Okumura, Rebecca Rosenke, Greg Saturday, Dana Scott, Heinz Feldmann

**Affiliations:** National Institutes of Health, Hamilton, Montana, USA (E. de Wit, F. Feldmann, E. Horne, C. Martellaro, E. Haddock, T. Bushmaker, K. Rosenke, R. Rosenke, G. Saturday, D. Scott, H. Feldmann);; Columbia University, New York, New York, USA (A. Okumura)

**Keywords:** Middle East respiratory syndrome coronavirus, MERS-CoV, animal models, domestic pig, dipeptidyl peptidase 4, DPP4, viruses, pigs, United States, zoonoses

## Abstract

We tested the suitability of the domestic pig as a model for Middle East respiratory syndrome coronavirus (MERS-CoV) infection. Inoculation did not cause disease, but a low level of virus replication, shedding, and seroconversion were observed. Pigs do not recapitulate human MERS-CoV and are unlikely to constitute a reservoir in nature.

As of March 10, 2017, a total of 1,917 cases of Middle East respiratory syndrome coronavirus (MERS-CoV) infection and 684 fatalities have occurred (*1*). Despite the relatively large number of cases, little is known about the disease pathology of MERS in humans (*2*). Our current understanding of the pathogenesis of MERS-CoV is therefore mostly based on data derived from studies in animal models. Although the first animal model used to study MERS-CoV pathogenesis and test potential countermeasures became available shortly after the discovery of MERS-CoV (*3*), all the animal models that have been developed so far have drawbacks (*4*). Because of the host restriction conferred by the binding of the MERS-CoV spike protein to its receptor, dipeptidyl peptidase 4 (DPP4), small animal models that are routinely used to conduct infectious disease research are not naturally susceptible to MERS-CoV infection. Although human DPP4-transgenic mouse models have been developed, these do not completely recapitulate the disease pathology observed in humans. Nonhuman primate models recapitulate mild and moderate human disease pathology; however, practical and ethical constraints limit work with these models.

The domestic pig (*Sus domesticus*) is used in infectious disease research because of similarities between human and pig anatomy, genetics, and physiology (*5*). MERS-CoV was previously shown to replicate in porcine kidney cells, albeit less efficiently than in human kidney cells (*6*). In an effort to develop a MERS-CoV animal model that recapitulates human disease better than small animal models without the constraints associated with nonhuman primate studies, we explored the possibility of using the domestic pig as an animal model of MERS-CoV infection.

## The Study

**Figure 1:**
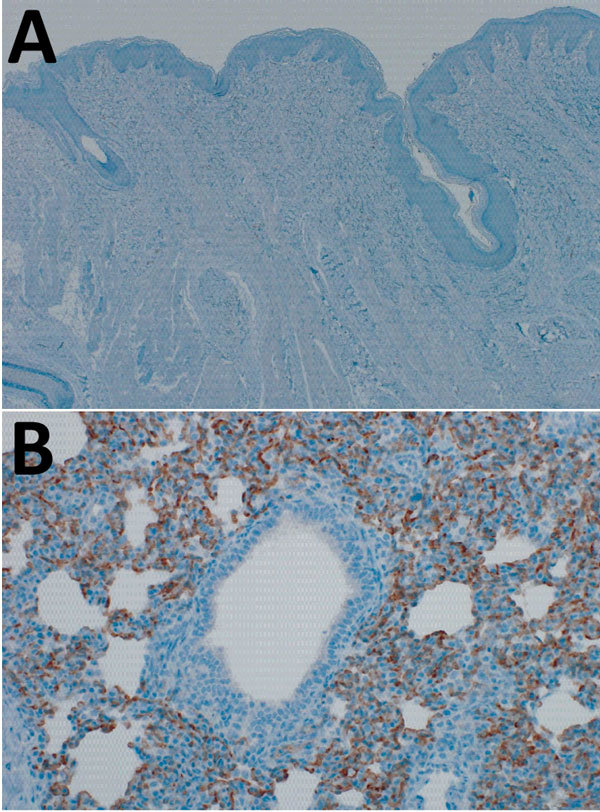
Dipeptidyl peptidase (DPP) 4 expression in the domestic pig respiratory tract. Tissues were stained by using a cross-reactive mouse monoclonal antibody against DPP4 (CD26, clone OTI11D7, 1:2,500; Origene Technologies, Inc., Rockville, MD, USA). DPP4 expression was absent in the nasal mucosa (A) but present in lung tissue (B) of healthy domestic pigs. Original magnification: nasal mucosa ×40; lung ×200.

Comparison of the DPP4 nucleotide sequences of humans, dromedary camels, and domestic pigs showed that the porcine DPP4 is identical to the dromedary camel DPP4 at the 14 aa positions that have been shown to determine species tropism (Table) (*8*,*9*). We investigated whether DPP4 is expressed in the pig respiratory tract by performing immunohistochemical staining on the nasal mucosa and lung tissue obtained from healthy pigs using an antibody against DPP4 (mouse monoclonal anti-DPP4 [CD26], clone OTI11D7, 1:2,500; Origene Technologies, Inc., Rockville, MD, USA). DPP4 expression was not observed in the nasal mucosa of healthy domestic pigs (Figure 1, panel A); in the lungs, abundant DPP4 expression was observed in type I and type II pneumocytes and submucosal glands (Figure 1, panel B), suggesting MERS-CoV infection would be supported.

**Table:** Comparison of the amino acid residues shown to be essential in binding of Middle East respiratory syndrome coronavirus spike protein to DPP4 of human, dromedary camel, and domestic pig*

Species	DPP4, aa position													
229	267	286	288	291	294	295	298	317	322	336	341	344	346
Human†	N	K	Q	T	A	L	I	H	R	Y	R	V	Q	I
Dromedary camel‡	–	–	–	V	–	–	–	–	–	–	–	–	–	–
Domestic pig§	–	–	–	V	–	–	–	–	–	–	–	–	–	–
Mouse¶	–	–	–	P	–	A	R	–	–	–	T	S	–	V

*DPP4, dipeptidyl peptidase 4; –, no change from human DPP4. †GenBank accession no. AB451339. ‡GenBank accession no. KF574263. §GenBank accession no. NM214257. ¶Taken from previous publication (*7*).

We inoculated 2 groups of four 4–5-week-old farm pigs (Yorkshire cross; S&S Farms, Ramona, CA, USA) intranasally (1 mL/nostril) and intratracheally (5 mL) with a total dose of 10^6^ tissue culture infectious dose 50 (TCID_50_) of the hCoV-EMC/2012 isolate of MERS-CoV. A group of 3 control pigs was mock inoculated with Dulbecco’s modified Eagle medium (DMEM); these pigs were housed in a separate room from the MERS-CoV–inoculated pigs to prevent cross-contamination. Animal experiments were approved by the Institutional Animal Care and Use Committee of the Rocky Mountain Laboratories and conducted by certified staff in an Association for Assessment and Accreditation of Laboratory Animal Care International–accredited facility according to the institution’s guidelines for animal use; staff followed the guidelines and basic principles in the US Public Health Service Policy on Humane Care and Use of Laboratory Animals and the Guide for the Care and Use of Laboratory Animals.

**Figure 2:**
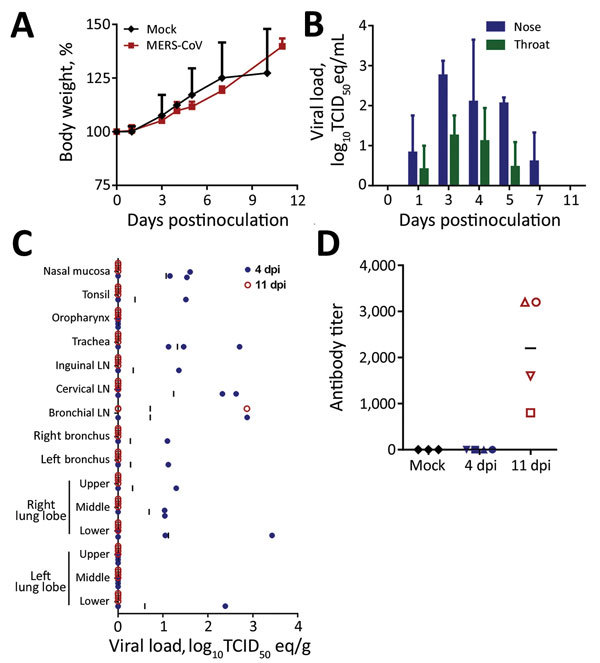
Propagation of Middle East respiratory syndrome coronavirus (MERS-CoV) in domestic pigs. We inoculated pigs intranasally and intratracheally with 10^6^ tissue culture infectious dose 50 (TCID_50_) of MERS-CoV isolate hCoV-EMC/2012 or, for controls (mock-inoculated), with Dulbecco’s modified Eagle medium. A) Mean bodyweight gain comparison between mock-inoculated and MERS-CoV–inoculated animals over time. Error bars indicate SDs. B) Mean viral loads shed from the nose and throat determined at the time points indicated by quantifying virus on nasal and throat swabs collected from MERS-CoV–inoculated animals using quantitative reverse transcription PCR (*11*); in each run, standard dilutions of a titered virus stock were run in parallel to calculate TCID_50_ equivalents. Error bars indicate SDs. C) Viral loads in tissues collected from MERS-CoV–inoculated animals on days 4 (closed blue circles) and 11 (open red circles) postinoculation. Viral loads were determined as in panel B. Vertical bars indicate means. D) Serum samples collected from pigs at the time of euthanasia (days 4 and 11 postinoculation) and tested for MERS-CoV antibodies by using an ELISA for MERS-CoV spike 1 protein. Antibody titers are plotted as the reciprocal of the last serum dilution positive by ELISA. Horizontal bars indicate means. LN, lymph node.

After inoculation with MERS-CoV, none of the pigs showed clinical signs of disease, such as increased body temperature or increased respiration, and bodyweight gain was similar between MERS-CoV–inoculated and mock-inoculated pigs (Figure 2, panel A). We collected nose and throat swabs during clinical exams and analyzed them for the presence of viral RNA by quantitative reverse transcription PCR (qRT-PCR) as described (*10*). Shedding of viral RNA from the nose and the throat increased from 1 day postinoculation (dpi) to 3 dpi in all MERS-CoV–inoculated animals, a sign that active replication occurred; shedding was higher in the nose than in the throat (Figure 2, panel B). After 3 dpi, shedding of viral RNA decreased; all nose swabs were negative by 11 dpi and all throat swabs by 7 dpi. 

We attempted virus propagation by inoculating VeroE6 cells with the media used to resuspend nasal and throat swab particulates and checking for the development of MERS-CoV cytopathic effect. Infectious MERS-CoV was not recovered at any time postinoculation from any swab sample.

On 4 and 11 dpi, we euthanized 4 MERS-CoV–inoculated pigs and collected their tissues for virologic and histologic analysis. Viral RNA could be detected by qRT-PCR in >1 respiratory tract tissue samples of all 4 MERS-CoV–inoculated pigs. However, viral loads were low; infectious MERS-CoV could not be isolated from any tissues positive by qRT-PCR, and the distribution of viral RNA among tissues was inconsistent from pig to pig (Figure 2 panel C). By 11 dpi, viral RNA could only be detected in the bronchial lymph node of 1 MERS-CoV–inoculated pig (Figure 2 panel C); all the tissues examined from other MERS-CoV–inoculated pigs were negative by this time. Viral RNA could not be detected in any of the extrarespiratory tissues tested, such as heart, liver, spleen, kidney, adrenal gland, duodenum, ileum, transverse colon, or urinary bladder, on 4 dpi or 11 dpi (data not shown). Histologic analysis did not reveal any lesions consistent with MERS-CoV infection in any of the collected tissues, including those of the respiratory tract. We performed immunohistochemical staining with an antibody specific for MERS-CoV on tonsil, trachea, bronchial lymph node, and right and left lower lung lobe of all pigs, as well as other tissues that tested positive for viral RNA by qRT-PCR. MERS-CoV antigen could not be detected in any of these tissues. 

Serum samples collected on the day of euthanasia were tested for the presence of antibodies against MERS-CoV spike protein 1 (S1) by ELISA. By 11 dpi, antibodies directed against MERS-CoV S1 could be detected in all 4 pigs (Figure 2, panel D).

## Conclusions

Recently, Vergara-Alert et al. showed MERS-CoV shedding in pigs inoculated with 10^7^ TCID_50_ of MERS-CoV and suggested that pigs could play a role as a reservoir for the circulation of MERS-CoV (*12*). In our hands, pigs inoculated with a 10-fold lower infectious dose of MERS-CoV were also successfully infected, but the low amount of virus replication in and shedding from the respiratory tract implies that the pig is unlikely to play a profound role as an intermediate host for MERS-CoV in nature.

Taken together, our data indicate that MERS-CoV can infect pigs, leading to a low level of replication in the pig respiratory tract, but does not cause clinical signs of disease. Furthermore, viral shedding from mucosal membranes of the upper respiratory tract was rather limited with no infectious virus measurable at any time postinoculation. Thus, the pig is not a suitable animal disease model for MERS-CoV infection. 
